# A Randomized Controlled Trial of an Intelligent Cognitive Stimulation Program for Adults with ADHD: Study Protocol

**DOI:** 10.3390/jcm14186629

**Published:** 2025-09-20

**Authors:** Elena Canadas, Fernando Maestu, Ignacio de Ramon

**Affiliations:** 1 Sincrolab, 28003 Madrid, Spain; nacho@sincrolab.es; 2 Center for Cognitive and Computational Neuroscience, Departamento de Psicología Experimental, Universidad Complutense de Madrid, 28015 Madrid, Spain; fmaestuu@eucm.es

**Keywords:** ADHD, Digital therapeutics, clinical trial protocol, Adult

## Abstract

**Background**: Attention-deficit/hyperactivity disorder (ADHD) often persists into adulthood, causing substantial functional impairments. While pharmacological treatments are considered first-line, many adults either decline or do not benefit from medication, underscoring the need for effective non-pharmacological interventions. **Objective**: This study describes the protocol for a randomized controlled trial (RCT) evaluating Sincrolab Adults, an AI-based cognitive stimulation program for adults with ADHD. The primary objective is to determine whether 12 weeks of intervention improves cognitive performance. Secondary and exploratory objectives assess its impact on ADHD symptoms, quality of life, and durability of effect. **Methods**: The study will employ a single-blind, multicenter, randomized controlled design comparing the digital cognitive intervention to a treatment-as-usual control group. A total of 104 adults diagnosed with ADHD will be randomized to either the Sincrolab Adults program or the control group. Outcomes will be measured using the MOXO Continuous Performance Test (CPT) for cognitive performance, the Adult ADHD Self-Report Scale (ASRS) for symptoms, and the Adult ADHD Quality of Life (AAQoL) scale. A one-month post-intervention follow-up will assess persistence of effects. **Results**: The primary outcome will be improvement in the CPT total score. Secondary outcomes include reductions in the ASRS symptoms. Exploratory outcomes will examine quality of life, ADHD symptom subdomains, and durability of improvements. **Conclusions**: This study will provide evidence on the feasibility and preliminary efficacy of a digital cognitive stimulation platform for adults with ADHD. Findings could support the development of scalable, accessible treatment alternatives for individuals with ADHD.

## 1. Introduction

Attention-deficit/hyperactivity disorder (ADHD) is among the most common childhood psychiatric disorders affecting 2.6–4.5% of children worldwide [1] and often persists into adulthood, with prevalence rates of 2–5% [2,3,4].

Adults with ADHD often experience significant psychosocial and functional challenges, including elevated levels of perceived stress [5], diminished academic and occupational productivity [6], and a greater likelihood of involvement in criminal behavior [7]. ADHD in adulthood also generates substantial healthcare costs [8]. A systematic review estimated that 89.5% of the global economic burden of ADHD falls on adults, while only 10.5% is attributed to children and adolescents [9]. In Spain, the average annual cost per adult is EUR 15,652, with an additional one-time cost of EUR 7893. These costs are primarily driven by economic factors (50%), social consequences (28%), healthcare spending (52%), and legal issues (42%) [10].

Pharmacological treatments, particularly stimulants, are considered first-line interventions for adults with ADHD [11] and have demonstrated efficacy in reducing core symptoms [12,13]. However, their impact on higher-order cognitive functions, such as executive functioning, working memory, or planning, remains limited, with many of these deficits persisting despite symptom improvement [14,15]. Moreover, these medications are frequently associated with side effects, including decreased appetite, abdominal pain, headache, irritability, insomnia, and increased anxiety. In rare cases, more serious psychiatric reactions such as mood disturbances or suicidal ideations have been reported, particularly in vulnerable individuals [16]. Consequently, many young adults discontinue medication to avoid dependence and adverse effects [17]. These limitations have contributed to increasing interest in non-pharmacological approaches, such as psychotherapy, psychosocial interventions, coaching, and skills training, as complementary or alternative approaches for managing ADHD in adults [18]. Guidelines from both the European Network Adult ADHD and the UK’s NICE recommend the use of psychological interventions, particularly cognitive behavioral therapy [19,20]. Cognitive training, a potentially valuable rehabilitation approach, has been minimally explored for adults with ADHD. Existing studies yield inconsistent results, possibly due to methodological variations or limitations. These interventions have demonstrated only modest improvements in cognitive performance in adults with ADHD, and evidence supporting their effect on core ADHD symptoms remains limited [21,22,23]. A recent meta-analysis found low-quality evidence for improvements in symptom severity, including reductions in inattention and hyperactivity/impulsivity. Additionally, the meta-analysis also highlighted the potential of digital interventions to enhance monitoring and patient–provider communication [24].

Neuroimaging studies implicate functional connectivity dysfunctions and neurocognitive deficits in cortical regions underlying impairment among individuals with ADHD [25]. These deficits manifest as deficits in sustained, selective, and divided attention [26], as well as in tasks related to executive functions, particularly inhibitory control, working memory, decision-making, and planning [27]. Additionally, deficits in processing speed and response time consistency have been observed [28]. Despite the promise of non-pharmacological treatments, their effects on cognitive rehabilitation remain limited [2,29].

Sincrolab Kids and Sincrolab Adults are innovative cognitive stimulation tools developed through collaboration among neuropsychologists, programmers, and video game designers. These tools employ gamified tasks grounded in neurorehabilitation principles and leverage the motivational and engaging aspects of video games. By immersing users in virtual environments that require integrated cognitive processing, these tools aim to promote the transfer of cognitive training to everyday tasks.

An artificial intelligence engine embedded in both tools dynamically adjusts task difficulty to maintain a performance level near 50%, ensuring individualized training programs tailored to each user’s abilities. Sincrolab Kids has undergone initial validation in a neurotypical population of 40 children aged 8–12, demonstrating significant improvements in inhibitory control and processing speed. A subsequent clinical trial with children diagnosed with ADHD-C incorporated behavioral assessments and neurophysiological measures via magnetoencephalography. Preliminary findings demonstrated significant improvements in visuospatial and phonological working memory, processing speed, and verbal fluency, along with enhanced functional connectivity between cortical regions [30,31,32], consistent with known neurocognitive correlates of ADHD [25].

Validation of cognitive stimulation tools like Sincrolab should follow an iterative and systematic process, tailored to the specific needs of different populations such as adults versus children with ADHD, individuals with varying symptom profiles, or those with other clinical conditions, including mild cognitive impairment [33]. Building on the successful validation of Sincrolab Kids for children and adolescents with ADHD, there is an urgent need to address the scarcity of scientifically validated non-pharmacological treatments for adults with ADHD. To this end, a randomized, single-blind, multicenter clinical trial is planned to evaluate the efficacy of Sincrolab Adults to expand therapeutic options for this underserved population.

## 2. Study Objectives

### 2.1. Primary Objective

The primary aim of the present study is to assess the efficacy of Sincrolab Adults in improving cognitive–functional performance in adults diagnosed with ADHD (any presentation) who are either not receiving pharmacological treatment or for whom medication has proven ineffective. The study aims to demonstrate that participants undergoing training with Sincrolab Adults exhibit significant improvements compared to those receiving a treatment-as-usual (control group). These improvements will be assessed using the MOXO Continuous Performance Test total score [34].

### 2.2. Secondary Objectives

The secondary aim is to assess whether treatment with Sincrolab Adults, compared to the control group, leads to significant changes in functional ADHD symptoms as measured using the Adult ADHD self-report Symptom Rating Scale (ASRS) [35]. Additionally, the study aims to evaluate whether these improvements reach clinically significant levels, defined as a minimum change of 12 points between baseline and post-treatment scores [36].

### 2.3. Exploratory Objectives

The exploratory aim is to (1) evaluate whether treatment with Sincrolab Adults, compared to the control, leads to significant improvements in quality of life, as measured with the Adult ADHD Quality of Life Questionnaire (AAQoL) [37]; (2) assess whether treatment with Sincrolab, compared to a control group, leads to significant improvements in the remaining indices from MOXO (attentiveness, timing, impulsivity, and hyper-reactivity); (3) to examine potential correlations between cognitive and functional outcomes—specifically, the study will investigate the relationship between CPT performance indices and functional symptoms and quality of life related to ADHD; and (4) to assess the durability of the effect of Sincrolab Adults following 1 month without intervention.

By addressing these objectives, this study aims to provide preliminary evidence for the feasibility and efficacy of Sincrolab Adults as a non-pharmacological intervention for adult ADHD, potentially offering a novel therapeutic approach for individuals who do not respond adequately to medication.

## 3. Materials and Methods

### 3.1. Design and Participants

This study is a parallel, randomized controlled trial (RCT) with a treatment-as-usual control group, employing a single-blind design to minimize bias. (Outcome assessors will be blinded to group allocation).

This study will include 104 adults aged 18 and older to evaluate Sincrolab Adults (v.4.0.24) (60% male/40% female, reflecting ADHD prevalence in Spain). The study will consist of adults with ADHD, with any presentation, who are stable on or off medication doses (for at least 2 weeks prior to baseline) and present persistent/stable symptoms (ADHD-RS ≥ 28). Participants will be divided into two equally sized groups (n = 52) that will either receive Sincrolab Adults or be placed in the control group.

The Sincrolab’s medical device consists of 14 tasks/games distributed across training sessions. Each training session includes a predetermined number of tasks, ensuring that different tasks are trained on each day. Each of the 14 tasks/games has a series of parameters that define them and allow for the identification of the cognitive processes involved (e.g., number of stimuli, number of distractors, stimulus speed, inter-stimulus interval, etc.). The intervention supports (a) the integration of various cognitive processes within each rehabilitation task/game; and (b) the adaptation of difficulty levels across the 14 tasks/games that make up the tool, based on each user’s performance in previous training sessions. The algorithms within the patented AiSA© artificial intelligence system adjust the difficulty level of cognitive stimulation tasks. Once AiSA has data on the participant’s performance during the initial cognitive training sessions, it uses this information to personalize the difficulty level of the tasks for each session and participant. In this way, cognitive training is completely personalized to each user.

The sample size per group in a two-sample design was calculated based on previous results [32] and using a moderate effect size (d = 0.62), as this provides a measure of the treatment effect. The required sample size will also assume a 20% dropout rate, resulting in 104 total participants, 52 per group.

The formula for the sample size per group in a two-sample design from Cohen (1988) [38] is used:

n = 2⋅(z_α/2_ + zβ)^2^/d^2^

where:

- z_α/2_: Critical value for the significance level (α = 0.05) = 1.96.
- zβ: Critical value for power (1 − β = 0.801) = 0.84.
- d: Effect size (d = 0.62).
- n: Sample size per group.

**Figure 1:**
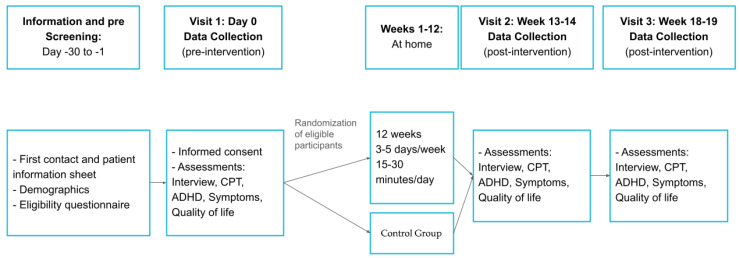
Study design showing participant flow, randomization, intervention with cognitive stimulation software, control group, and assessment time points.

The study protocol (Figure 1) will span 12 weeks plus an additional month of follow-up, with three assessments (baseline, post-intervention, and follow-up). Participants in the intervention group will use a digital cognitive training program designed to target multiple cognitive functions associated with ADHD dysfunctions. The program will include adaptive training games that can be played on a computer or mobile device. Participants will play the training games 3 to 5 times per week, for 15 min per session, over a 12-week period. Participants in the control group will not receive the intervention during the 12-week trial but will be offered access to the program after the study concludes.

The study flow diagram is as follows:

Inclusion criteria.

Participants must meet all of the following:

○ Adults aged 18 and older
○ Clinical diagnosis of ADHD according to DSM-5 criteria, confirmed using the DIVA 5.0 Interview for Attention-Deficit/Hyperactivity Disorders Studies (Adult version)
○ Stable use or non-use of ADHD medications for at least 2 weeks prior to enrollment and throughout the 12-week study period
○ Baseline ADHD-RS score ≥ 28
○ Ability to understand and follow written and verbal instructions in Spanish, as assessed by the Principal Investigator or study coordinator
○ Completion of the informed consent form
○ Access to a compatible digital device
○ Willingness and ability to comply with all study procedures and testing requirements

Exclusion criteria.

Participants meeting any of the following will be excluded:

○ Co-occurring neurological disorders or severe psychiatric conditions (e.g., schizophrenia, bipolar disorder)
○ Suicidality, as assessed using the Columbia-Suicide Severity Rating Scale (C-SSRS)
○ Motor impairments (e.g., physical deformities of the hands/arms) preventing gameplay, as reported by the participant or observed by the investigator
○ History of moderate or severe substance use disorder within the 12 months prior to informed consent
○ History of seizures (excluding febrile seizures), significant tics, or current diagnosis of Tourette’s Disorder
○ Known sensitivity to video games (e.g., photosensitive epilepsy, lightheadedness, dizziness, nausea, or motion sickness)
○ Any medical condition that, in the opinion of the investigator, may confound study data or assessments
○ Planned initiation of or significant changes in non-pharmacological behavioral therapy during the study
○ Planned initiation of or significant changes in non-pharmacological training (e.g., game/app-based cognitive training or neurofeedback) during the study

### 3.2. Withdrawal

Participants who choose to withdraw from the study for reasons not defined in the research protocol may do so without needing to justify their decision. However, the potential causes for withdrawal are anticipated to include the following: (1) Inability to comply with the training protocol due to scheduling conflicts, (2) the occurrence of an adverse event in the participant’s family, and/or (3) the occurrence of adverse events affecting the participant.

When a participant notifies their intention to withdraw from the study, a follow-up call will be made to encourage compliance. If this is not possible, an attempt will be made to obtain feedback regarding the reason for their withdrawal from the study.

### 3.3. Treatment Allocation

Participants will be randomly assigned to treatment groups (experimental vs. control). Both groups will undergo the same evaluations: CPT, ADHD symptoms, and Quality of Life questionnaires. This will be a single-blind study. The evaluators (assessors and data analysts) will remain blind to the experimental condition of the participants, which will only be revealed after the final visit. At that point, control group participants will have the option to receive the treatment with the same duration of intervention as the experimental group.

### 3.4. Ethics and Registration

This study will adhere to the ethical standards set forth by the Declaration of Helsinki (Edinburgh, 2000), ISO standard 14155 [39], and all other applicable device and Spanish regulations. Approval from the appropriate institutional review board (IRB) will be obtained with informed consent collected from all participants. Researchers and therapists will closely monitor participants to prevent clinical deterioration, and post-treatment assessment will capture any adverse or unwanted events.

### 3.5. Statistical Analysis

The primary analysis will employ linear mixed-effects models (LMMs) to compare changes in MOXO CPT total score over time between groups. Fixed effects will include treatment group, time, and their interaction, with random intercepts for participants to account for within-subject variability. Demographic factors such as age, gender, and ADHD presentation will be included as covariates.

Secondary analyses will follow a similar approach, using generalized linear mixed models (GLMMs) for outcomes like ASRS scores and LMMs for continuous measures of cognitive performance. Clinically significant improvements in ADHD symptoms, defined as a ≥12-point reduction in ASRS scores, will be analyzed using logistic regression.

Exploratory analyses will assess the durability of treatment effects over a 1-month follow-up period, using additional time points in the mixed-effects models. Correlations between cognitive and functional measures, particularly CPT indices (e.g., omissions and concentration) and ASRS scores, will be explored using Pearson or Spearman correlation coefficients, with the false discovery rate (FDR) method applied to control for multiple comparisons.

To address potential missing data, logistic regression will be used to determine whether missingness is random or systematic. Multiple imputation methods [40] will be employed to account for missing values, and sensitivity analyses will be conducted to compare complete-case results with those from imputed datasets, ensuring robustness. Model selection will be guided by the Bayesian Information Criterion (BIC), and diagnostics will include residual checks to validate model assumptions.

All analyses will be conducted using R (version 4.3.1, 2025) or JASP (version 0.19.1, 2024). For linear mixed-effects models, we will use the lm4 package in R; for generalized models, the glmmTMB package will be applied. Missing data will be handled using the mice package (Mice 3.16.0) for multiple imputation. Residual diagnostics and model assumptions will be evaluated using performance and DHARMa. Correlation analysis will be conducted using base R or psych (R 4.5.0; Psych package 2.5.), with *p*-values adjusted using the Benjamini–Hochberg method to control the false discovery rate. This comprehensive approach will ensure robust evaluation of the efficacy and potential long-term benefits of Sincrolab Adults, while exploring associations between cognitive and functional outcomes in adults with ADHD, thereby assessing the value of Sincrolab as a non-pharmacological treatment option.

### 3.6. Expected Outcomes

Results will be reported in accordance with the CONSORT 2010 guidelines.

The primary outcome will be reported as the change from baseline to post-intervention in MOXO CPT total score, presented as estimated marginal means with standard errors for each group, along with 95% confidence intervals and effect sizes (Cohen’s d). It is anticipated that participants in the Sincrolab Adults group will show greater improvement compared to the control group.

Secondary outcomes (e.g., ASRS scores) will be reported as mean changes, proportions of participants achieving clinically significant improvement (defined as a reduction of 12 points or more on ASRS), and corresponding odds ratios and confidence intervals. It is expected that the Sincrolab Adults group will exhibit a greater reduction in ASRS score and a higher proportion of participants achieving a clinically significant improvement compared to the control group.

Exploratory outcomes (AAQoL scores, additional MOXO indices) will be presented as mean (±standard deviation) pre–post changes for each group, along with group × time interaction *p*-values and effect sizes for each exploratory measure. Participants in Sincrolab Adults are expected to show greater pre–post gains in quality of life and MOXO sub-indices compared with controls. We anticipate positive correlations between improvements in CPT indices and improvements in functional outcomes (ASRS) and quality of life (AAQoL). Finally, we expect treatment benefits to be maintained at the 1-month follow-up.

## 4. Discussion

This study describes a clinical trial designed to evaluate the efficacy of Sincrolab Adults, an AI-based cognitive training intervention tailored for adults with ADHD (inattentive or combined presentation). While pharmacological treatments remain the standard of care, many adults with ADHD do not respond adequately to medication or discontinue due to side effects. Cognitive training offers a promising, non-pharmacological alternative, yet evidence supporting its effectiveness in adults remains limited. This study aims to address this gap in the literature.

Digital cognitive training is theoretically grounded in the concept of experience-dependent neuroplasticity [41], whereby repeated, adaptive cognitive challenges strengthen neural circuits involved in attention, working memory, and inhibition—core deficits in ADHD. Adaptive training that dynamically adjusts task difficulty may enhance engagement and target individual cognitive limitations more effectively than fixed protocols. Improvements in these cognitive domains (i.e., near-transfer effects) may translate into better performance on tasks like the MOXO-CPT, which relies on sustained attention, impulse control, and processing speed. Additionally, gains in these areas may produce far-transfer effects, such as reductions in daily ADHD symptoms (ASRS) and improvements in quality of life (AAQoL). However, such generalization remains a matter of empirical debate [16,23].

This study may also provide insight into the broader accessibility and public health potential of online cognitive interventions. Digital platforms like Sincrolab Adults have the capacity to overcome geographical, economic, and logistical barriers that limit access to conventional, face-to-face therapies. For individuals from underserved or socioeconomically disadvantaged backgrounds, these tools could offer a scalable and cost-effective alternative, particularly in regions with limited access to adult ADHD specialists. However, issues of digital equity remain an important consideration. Future research should examine how factors such as internet access, digital literacy, and cultural context influence engagement and uptake across diverse populations.

It is also important to consider that observed improvements on performance-based tasks may be influenced by practice effects, and changes in self-reported outcomes could partly reflect expectancy biases, especially given the use of a treatment-as-usual control rather than an active placebo. While outcome assessors and data analysts are blinded to group allocation to reduce detection and analysis bias, participants are aware of their assignment, which may affect subjective reporting. These limitations will be carefully considered when interpreting the findings and will be addressed through sensitivity analyses where appropriate.

Additionally, this trial will explore potential moderators such as ADHD subtype (inattentive vs. combined), medication status, adherence to training, and use intensity. These variables may help identify individuals most likely to benefit from cognitive training. The inclusion of a 1-month follow-up will also provide preliminary insight into the durability of treatment effects, which remains a critical issue in digital health interventions.

### 4.1. Strengths

One of the primary strengths of this study is its personalized, adaptive treatment approach. Sincrolab Adults utilizes a series of adaptive cognitive training games that dynamically adjust to match each participant’s individual cognitive profile. This level of personalization is expected to enhance engagement and treatment adherence, potentially leading to greater efficacy than conventional, non-adaptive interventions.

A key strength of this study is the single-blind design, in which both outcome assessors and data analysts are blinded. This approach minimizes detection and analysis bias, enhancing the reliability and validity of objective cognitive and functional outcomes. Although patients are aware of their group assignment and placebo or performance effects cannot be fully controlled, the design ensures robust assessment of treatment effects while maintaining internal validity. Additionally, the research team, including Sincrolab, has extensive experience in digital therapeutics (DTx) research and ADHD clinical studies, ensuring high methodological rigor in recruitment, treatment delivery, data collection, and analysis. The expertise of the investigators and the established research infrastructure contribute to the feasibility and successful execution of this trial.

### 4.2. Limitations and Future Directions

Despite its strengths, the study also presents several limitations that should be acknowledged. One notable limitation is the relatively short follow-up period and the limited number of measurement points. While additional assessments during the intervention period could provide a more granular understanding of treatment effects over time, the practical challenges associated with repeated testing in adults with ADHD, such as inattention, restlessness, and difficulties with sustained engagement, necessitate a parsimonious approach. To maximize participant retention in follow-up assessments, reminders will be systematically sent, and study personnel will actively reach out to non-responders.

Another potential challenge is participant attrition throughout the intervention. Given the well-documented executive functioning difficulties in ADHD, dropout rates may be higher than those observed in other clinical populations. To mitigate this risk, participants who fail to complete training modules will receive weekly follow-ups, along with technical and motivational support as needed.

Furthermore, although adherence metrics (e.g., the number of completed training sessions and total time spent on tasks) will be monitored, an inherent limitation remains in quantifying the quality of engagement during training sessions. While adherence data provide a proxy for treatment exposure, they do not capture variability in motivation, attentional effort, or strategy use, which may influence outcomes. Future studies may benefit from incorporating real-time monitoring or behavioral analytics to assess engagement quality more precisely.

Importantly, while outcome assessors will be blinded to group allocation, participants themselves will not, due to the nature of the intervention. This introduces the possibility that expectancy effects could influence self-reported outcomes. Additionally, although the use of a control group is ethically and practically appropriate, it does not account for placebo effects or the influence of structured engagement, factors that may contribute to observed improvement.

Finally, since the study will be conducted within a specific healthcare and cultural context, the generalizability of findings may not be fully generalizable to other populations or health systems.

Future research should consider the inclusion of active control groups (e.g., sham training or non-specific digital tasks) to better isolate the specific effects of cognitive stimulation. Studies with longer-term follow-up and more frequent assessments would provide greater insight into treatment durability and the timing of clinical response. Additionally, expanding recruitment to more diverse settings and populations would enhance the external validity of the findings. Finally, incorporating objective measures of engagement and behavioral responses may help clarify the mechanisms underlying individual differences in treatment outcomes.

## 5. Conclusions

Despite these limitations, this study has the potential to make a meaningful contribution to the field of ADHD treatment by evaluating a digital cognitive training intervention with a rigorous, controlled methodology. Given the limited availability of validated non-pharmacological treatments for adults with ADHD, this research could help address a critical gap in care. If successful, the findings could support the broader implementation and dissemination of digital therapeutics for ADHD, ultimately expanding access to evidence-based interventions for individuals who may not respond adequately to medication. Future studies should focus on refining digital cognitive training approaches, assessing longer-term outcomes, and exploring how these interventions can be applied in real-world clinical settings across diverse populations.

## Data Availability

No data are available at this time. Data will be published and shared upon request once available.
